# Dairy farming systems driven by the market and low-cost intensification in West Africa: the case of Burkina Faso

**DOI:** 10.1007/s11250-021-02725-z

**Published:** 2021-04-26

**Authors:** Eric Vall, Ollo Sib, Arielle Vidal, Jethro Barkwende Delma

**Affiliations:** 1 SELMET, CIRAD, UMR SELMET, F-34398 Montpellier, France - SELMET Univ Montpellier, CIRAD, INRA, Montpellier SupAgro, Montpellier, France; 2 Département des Sciences Animales, Agro-Alimentaires, Nutrition et Santé, Universite de Toulouse, Ecole dIngenieurs de Purpan, 75 voie du TOEC, BP 57611, 31076 Toulouse, France; 3 CNRST, INERA, Saria, Burkina Faso

**Keywords:** Dairy farming systems, Intensification, Market, Agroecology, Africa

## Abstract

The increase in demand for dairy products in Burkina Faso is encouraging livestock producers to develop milk production. Three types of dairy systems (pastoralists, agropastoralists and market-oriented dairy farms) have been characterised based on a sample of 60 producers operating in the West and centre of the country. Pastoralists’ dairy operations consist mainly of zebus, rely on pasture for feed, store little fodder, and recover little manure. Milk yields are low (1.4 l/tropical livestock unit (TLU)/day) and milk sales are limited, but mostly benefit women. Agropastoralists’ dairy operations consist mainly of zebus, store more fodder for feed, use more concentrate and recover manure better. Milk yields are higher (3.1 l/TLU/day) and milk sales are threefold those of pastoralists, but less of the money generated by milk sales goes to women. Market-oriented dairy farmers’ operations are mainly made up of crossbreds, reared indoors and fed on fodder and feeds, store much more fodder and recover manure even better. They generate the highest milk yields (7.3 l/TLU/day), and milk sales are 2.5-fold those of agropastoralists. However, money earned from milk sales mainly benefits men. The study shows that the improvement in dairy systems’ technical and economic performance, which mostly rests on genetics and cow feed, but also on better recycling of agricultural by-products, is driven by a low-cost intensification and market opportunity (raising processors demand).

## Introduction

While still low (~14 l/capita/year in 2018 according to the FAO), milk consumption in West Africa is increasing sharply. Milk production rose substantially between 2000 and 2018 (~+87% according to the FAO) and is estimated at around 6 million tonnes of litres per year, 60% of which comes from cows (Corniaux et al. 2014). As a result, more and more livestock producers in Burkina Faso increase milk production to supply the domestic market (Sib et al. 2017; Vidal et al. 2020). This raises the issue of productivity and sustainability of those emerging dairy operations.

For a long time, livestock producers in Burkina Faso seemed to have steered clear of the milk market, as shown by the dairy farmer typologies of Hamadou et al. (2003) and Ouédraogo (1995) established on the outskirts of Bobo-Dioulasso and Ouagadougou, respectively. These typologies revealed two groups of livestock producers based on the degrees of production intensification and integration to the market. According to these studies, the first and overwhelmingly largest group (98.5%) consisted of pastoralists and agropastoralists who saw milk production as a secondary economic activity after cattle trading. These holdings were characterised by the breeding of zebu cows, with pasture as the main feed resource, a very low milk yield per heifer, and limited marketing of the milk produced. The second group, much smaller (1.5%), consisted of dairy farmers who were in the process of specialisation and were market-oriented. For them, milk production was the main objective. Consequently, in order to secure their activity and increase production, they were pursuing a land acquisition strategy and developing more intensive dairy farming practices (i.e. use of feed concentrates, exotic local breeds and sometimes artificial insemination). Recent studies by Sib et al. (2017) and Vidal et al. (2020) in the Bobo-Dioulasso area, and by Gnanda et al. (2016) on the outskirts of Ouagadougou, have shown that the dairy production landscape in Burkina Faso is still dominated by the same categories of producers, but with an increasing proportion of market-oriented dairy farmers. The primary objective of this study is to characterise the different dairy systems more accurately than in previous studies by providing the most accurate quantifying elements possible. The ultimate objective is to compare dairy farmers’ practices at dairy system level, assess the performance of dairy systems and provide information on drivers and sustainability of ongoing developments within dairy systems.

## Materials and methods

The survey was based on a sample of 60 milk-producing farms representative of the range of types highlighted by previous studies (Hamadou et al. 2003; Gnanda et al. 2016; Sib et al. 2017; Vidal et al. 2020): pastoralists, agropastoralists and market-oriented farms, spread between 30 holdings in the Kadiogo province (Ouagadougou; 12° 22′ 17.14″ N, 1° 31′ 10.78″ W), 22 in the Houet (around Bobo-Dioulasso; 11° 11′ 9.78″ N, 4° 16′ 58.68″ W), 5 in the Tuy (around Koumbia; 11° 13′ 53.97″ N, 3° 41′ 20.52″ W) and 3 in the Comoé (around Banfora; 10° 38′ 24.06″ N, 4° 45′ 31.70″ W) provinces.

Data was collected through a once-through survey questionnaire, applied to each farm manager considering 1 year of production (from February 2018 to January 2019), focusing on the dairy operation (during the hot dry season (from February to May 2018), then during the rainy season (from June to October 2018) and then during the cold dry season (from November 2018 to January 2019)). A principal component analysis (PCA) was applied to twenty informed and calculated variables, followed by an ascending hierarchical classification (AHC) in order to distinguish uniform classes. Those twenty variables included:

- Structure-related variables: the cattle herd was estimated from the count of heads carried out during the survey and was expressed in tropical livestock unit (TLU); 1 TLU=adult weighing 250 kg; and considering 1.5 TLU for an adult male zebu and a crossbred dairy cow, 1 TLU for an adult dairy zebu cow, 0.8 TLU for a young bull and a heifer and 0.4 TLU for a calf; crops (ha, including fodder crops); number of milking cows (in TLU; i.e. Sudanese Fulani zebu plus cross-bred milking cows); and percentage of cross-bred milking cows (%; cross-bred issued from artificial insemination (AI) zebu x Holstein, or zebu x Montbéliarde, or zebu x Brune des Alpes, or zebu x Tarentaise)
- Operating variables: pastured ingested, fodder ingested and feed ingested per milking cow (in kg of dry mater (DM)/TLU/day, considering 6.25 kgDM/TLU/day for a full 14 h day of grazing); fodder stored (kg/TLU/year); manure lost on pasture (kg/TLU/year); and manure recovered for farm needs (kg/TLU/year)
- Performance variables: average milk yields (l/TLU/day), milk sold (l/TLY/year), milk production costs in CFA F/TLU/year; 1 euro = 655,957 CFA F) for wages, for feeds and for healthcare; income from milk and profit margin (CFA F/TLU/year); and milk income managed by women of the household (yes or no)

We carried out an ANOVA test completed by a Newman-Keuls test to see whether differences between classes were significant at the 5% threshold.

## Results

**Fig. 1:**
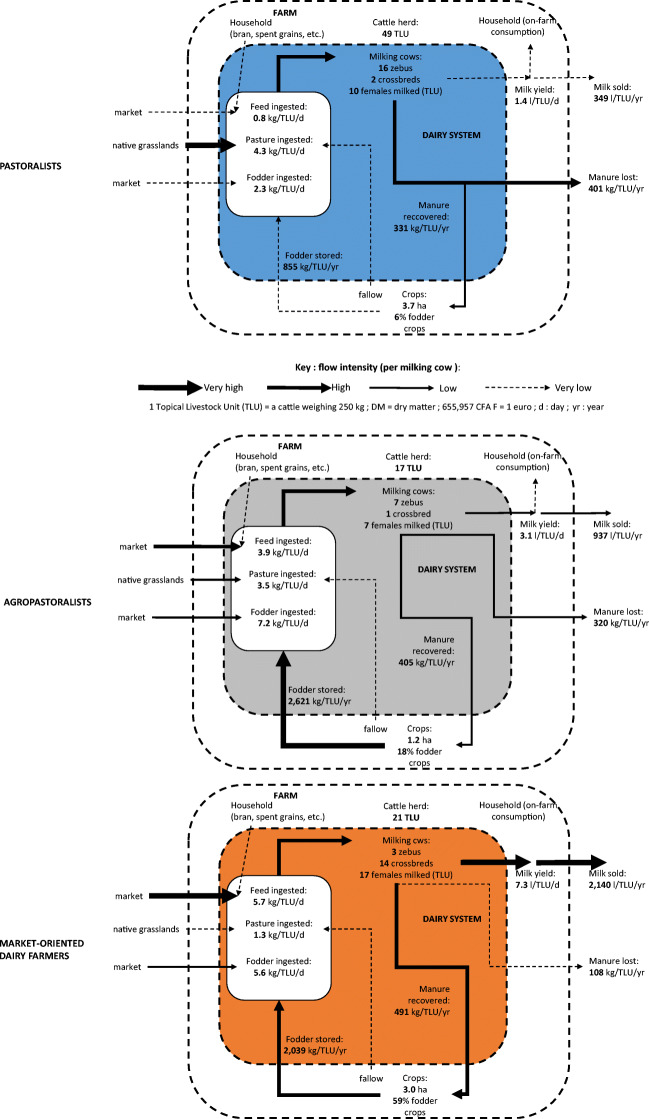
Dairy systems of the three classes of producers

The PCA and the AHC brought out three classes of dairy systems (Table 1; Fig. 1).

**Table 1:** Means (standard deviations), results of the ANOVA and Newman-Keuls tests according to the three dairy system classes

Type of dairy systems	Pastoralists (P)	Agropastoralists (AP)	Market-oriented Dairy farmers (F)	ANOVA Pr > F
Cattle herd (TLU)	48.7^a^ (14.8)	17.0^b^ (7.0)	21.0^b^ (5.5)	< 0.0001
Crops (ha)	3.7^a^ (2.0)	1.2^b^ (0.9)	3.0^a^ (1.9)	0.001
Milking cows (TLU)	9.8^b^ (4.1)	6.6^c^ (2.4)	17.4^a^ (5.5)	< 0.0001
Crossbred milking cows (%)	11^b^ (16)	8^b^ (20)	78^a^ (27)	< 0.0001
Fodder crop (% of cultivated area)	6^b^ (6)	18^b^ (34)	59^a^ (40)	< 0.0001
Pasture ingested (kgDM/TLU/d)	4.3^a^ (0.8)	3.5^b^ (1.1)	1.3^c^ (1.5)	< 0.0001
Fodder ingested (kgDM/TLU/d)	2.3^b^ (1.5)	7.2^a^ (3.9)	5.6^a^ (3.1)	< 0.0001
Feed ingested (kgDM/TLU/d)	0.8^c^ (1.1)	3.9^b^ (2.4)	5.7^a^ (3.4)	< 0.0001
Fodder stored (kg/TLU/yr)	854.9^b^ (561.4)	2621.0^a^ (1423.9)	2039.3^a^ (1119.6)	< 0.0001
Manure lost (kg/TLU/yr)	400.8^a^ (73.1)	320.3^b^ (105.6)	108.2^c^ (131.0)	< 0.0001
Manure recovered (kg/TLU/yr)	330.9^c^ (45.1)	405.4^b^ (57.0)	491.2^a^ (70.4)	< 0.0001
Wage costs (CFA F/TLU/yr)	3704^c^ (5651)	55559^a^ (44153)	35190^b^ (12202)	< 0.0001
Milk yield (l/TLU/d)	1.4c (0.8)	3.1b (1.2)	7.3a (2.5)	< 0.0001
Milk sold (l/TLU/yr)	349.3c (327.8)	936.8b (278.3)	2139.8a (642.7)	< 0.0001
Feed costs (CFA F/TLU/yr)	24216^b^ (32190)	196959^a^ (145697)	251292^a^ (120 761)	< 0.0001
Healthcare costs (CFA F/TLU/yr)	6525^a^ (4224)	2583^b^ (1388)	3237^b^ (2702)	0.000
Households where women manage milk income (%)	60^b^	40^b^	18^a^	0.000
Income from milk (CFA F/TLU/yr)	63602^c^ (57896)	305500^b^ (95682)	798142^a^ (297 997)	< 0.0001
Profit margin (CFA F/TLU/yr)	29156^b^ (32730)	50398^b^ (164523)	508423^a^ (303 282)	< 0.0001
Milk costs (CFA F/l)	115.1^b^ (62.8)	292.8^a^ (234.5)	124.5^b^ (50.2)	0.000

Key: 1 tropical livestock unit (TLU) = a cattle weighing 250 kg; DM = dry matter; 655,957 CFA F = 1 euro; *d* day, *yr* year

*Pastoralists’ dairy operations* feature low milk yields (1.4 l/TLU/day) and low volumes of milk sold (349 l/TLU/year). Pastoralists have large cattle herds (49 TLU). Their dairy operation is largely dominated by Sudanese Fulani zebus (16 zebus and only two crossbred milking cows: zebu x Holstein, or zebu x Montbéliarde, or zebu x Brune des Alpes depending of the farms). On average, they milk 10 milking cows per day. They make extensive use of grazing (native grasslands ingested estimated at 4.3 kgDM/TLU/day). Pastoralists provide very little fodder and feed rations (2.3 and 0.8 kgDM/TLU/day). They store very few crop residues for fodder (855 kgDM/TLU/year). They cultivate small areas (3.7 ha/farm: mainly maize and sorghum) and grow little fodder (6% of the cultivated area; cowpea or velvet beans). Faeces recycling is low. The amount of manure recovered for farm needs is estimated at 331 kg/TLU/year, while the quantity of manure lost on pasture is 401 kg/TLU/year. They have the lowest production costs (34,446 CFA F/TLU/year for wages + feeds + healthcare; 115 CFA F/l). Costs can be broken down as follows: feed 70%, healthcare 19% and wages 11%. Income from milk sales and profit margin on milk are the lowest among the three classes, at 63,602 CFA F/TLU/year and 29,156 CFA F/TLU/year, respectively. For pastoralists, milk production remains a minor economic activity for the household as a whole but primarily benefits women in 60% of cases.

*Agropastoralists’ dairy operations* are characterised by higher milk yields (3.1 l/TLU/day) and higher volumes of milk sold (937 l/TLU/year) than those of pastoralists. Agropastoralists have smaller cattle herds (17 TLU). On average, they milk 7 cows per day, mostly Sudanese Fulani zebus (7 zebus and 1 crossbred: zebu x Holstein, or zebu x Montbéliarde, or zebu x Brune des Alpes). They use pasture to feed their milking cows, but less intensely than pastoralists (pasture ingested on native grasslands was estimated at 3.5 kgDM/TLU/day). However, they provide larger fodder and feed rations, at 7.2 and 3.9 kgDM/TLU/day, respectively. They store more crop residues than pastoralists for fodder (2621 kg/TLU/year). Their cultivated area is smaller (1.2 ha/farm: mainly maize and sorghum), and they grow more fodder (18% of the cultivated area; cowpea or velvet beans). They recycle faeces more efficiently than pastoralists (405 kg/TLU/year of manure recovered for farm needs and 320 kg/TLU/year of manure lost on pasture). Given their higher level of production intensification, agropastoralists have higher costs than pastoralists (255,101 CFA F/TLU/year for wages + feeds + healthcare; 293 CFA F/l of milk). Their highest cost item is feed (77%), followed by wages (22%), with healthcare costs only amounting to 1%. Income from milk sales and profit margin on milk are significantly higher than for pastoralists, at 305,500 CFA F/TLU/year and 503,980 CFA F/TLU/year, respectively. Within the household, milk plays a greater economic role than among pastoralists, but the proportion of women benefiting directly from the milk income falls to 40%.

*Market-oriented dairy farmers’ operations* feature the highest milk yields (7.3 l/TLU/day) and a significantly higher volume of milk sold than pastoralists and agropastoralists (2140 l/TLU/year). They deliver most of their milk to a dairy. Their cattle herds are of medium size (21 TLU). They milk an average of 17 milking cows per day. Their dairy operation consists mainly of Sudanese Fulani zebu crossed by artificial insemination with exotic dairy breeds (Holstein, Brune des Alpes or Montbéliarde or Tarentaise; 14 crossbred cows and 3 zebus). They are less reliant on grazing (pasture ingested on native grasslands is estimated at 1.3 kg/TLU/day). They provide similar fodder rations to those of agropastoralists, but larger than those of pastoralists (fodder ingested estimated at 5.6 kgDM/TLU/day). They make greater use of feed concentrates (cottonseed meal or corn bran) than the other classes (feed ingested estimated at 5.7 kgDM/TLU/day). Some farmers provide excessive amounts of concentrates. Like agropastoralists, they store large quantities of crop residues for fodder (2039 kg/TLU/year). Their cultivated area is similar to that of pastoralists (3.0 ha/holding: mainly sorghum and secondly maize), but they grow more fodder (59% of the cultivated area; mainly forage sorghum which is often processed into silage and secondly cowpea). Faeces recycling is more efficient than among pastoralists and agropastoralists (491 kg/TLU/year of manure recovered for farm needs and only 108 kg/TLU/year of manure lost on pasture). Due to the intensification of production through labour and the use of feed concentrates, their costs are the highest among all three classes (289,719 CFA F/TLU/year for wages + feeds + healthcare; 125 CFA F/l of milk). Their costs are similar to those of agropastoralists, with an increase in feed costs due to the purchase of concentrates: feed (87%), wages (12%) and healthcare (1%). They also boast the highest levels of income from milk sales and the highest profit margins on milk, at 798,142 CFA F/TLU/year and 508,423 CFA F/TLU/year, respectively. For these producers, milk production is often a major economic activity, the income of which is mostly managed by men (82% of cases).

## Discussion

The study shows that the improvement in dairy systems’ technical and economic performance mainly rests on cow genetics, their feed and a better recycling loop for agricultural by-products. It also shows that women are sadly being excluded from the income generated from milk as dairy activity becomes more important in the household. These four points serve as a framework for our discussion aimed at analysing this ongoing transition from an efficiency and sustainability perspective.

### Changes in breeding and reproduction practices

Production levels per milking cows in the study area (500 to 2500 l/lactation) are similar to those recorded in the savannah and Sahel regions (Morin et al. 2007; Gaye et al.., 2020) but remain below those achieved in tropical Highland areas (Bebe et al. 2003) and in areas with more intensive livestock farming (Ageeb and Hayes 2000; Kahi et al. 2000). This is because dairy systems mostly involve local zebus (Sudanese Fulani zebus) not selected for their dairy output. In the Highlands of Kenya, 78% of small dairy producers rear exotic dairy cattle for their high milk yield, often in stalls with little access to pasture, and only 22% rear local zebus (Bebe et al. 2003). In West Africa and especially in Burkina Faso (Morin et al. 2007; Gaye et al. 2020), the interest in exotic breeds and artificial insemination (AI) is growing but remains limited. These practices can sometimes have limitations in a farming environment when they are not properly managed (risk of inbreeding). They also raise serious animal welfare issues when it comes to breeds ill-suited to tropical heat (Ageeb and Hayes 2000; Kahi et al. 2000), and AI protocols are subject to debate owing to the conditions under which hormones are produced (Grimard et al. 2003). The AI of local zebu (Sudanese Fulani) with exotic dairy breeds (Montbéliarde, Holstein, Brune des Alpes, Tarentaise) increase quickly milk production, which is appreciated by dairy farmers. However, to stabilise this progression over time, it is essential to supplement AI with programs for the selection of promising heifers and young bulls among the offspring of crossed dairy mothers, in order to sustainably increase among the local cows of tomorrow the milk yield (around 10 l/day; improvement conveyed by exotic dairy breeds) while maintaining their adaptation to the hot climate of the savannahs (character provided by the hardiness of the local zebu).

### Changes in feeding practices

The role of pasture in feed rations declines as milk becomes more economically important to the holding, unlike fodder and feed concentrates (Table 1). For instance, among market-oriented dairy farmers, grazing is significantly reduced in comparison to pastoralists and agropastoralists but nevertheless remains an important part of the ration. In both western and eastern Africa, reduced grazing coupled with a stronger commitment to milk production and trade is a general trend resulting in the adoption of stalling (Bebe et al. 2003; Morin et al. 2007; Gaye et al. 2020). In both regions, fodder mostly consists of crop residues collected from the field and stored. Unlike East Africa, silage and fodder crops are still in their infancy in West Africa (Njarui et al. 2011), except in some peri-urban areas such as the outskirts of Ouagadougou. After genetics, feed concentrates are very widely used by producers as a way of increasing production. In the semi-arid zones of Kenya, Njarui et al. (2011) found that 88 to 92% of dairy farmers provided feed at a rate of 2 kgDM/cow/day on average. In Burkina Faso, market-oriented dairy farmers tend to provide feed in abundance to the milking cows (5.7 kgDM/TLU/day) as they are inexpensive and seen as a simple and reassuring way of increasing milk yields. However, such excessive use is neither efficient nor without risks (acidosis).

### Changes in the practice of combining crop and livestock farming

The study shows that the increase in cultivated fodder (mainly cowpea and velvet bean hay or silage of forage sorghum in this case study and possibly grass forages such as *Brachiaria* sp. or shrub fodder such as *Leucaena* sp. or *Albizia* sp.; Sib et al. 2017) used in rations goes hand-in-hand with better recycling of cultivated crop residues (maize and sorghum straw) and animal faeces (Table 1). This leads to greater self-sufficiency in fodder and manure for dairy farmers, more efficient use of resources and preservation of soil fertility, i.e. improved sustainability of the holding. This can partly be explained by strong land pressure in peri-urban areas, where dairy farmers who deliver to dairies are established. In the Highlands of Kenya, Udo et al. (2011) showed that, due to increased land pressure, more than three-quarters of dairy farmers intensified their farming practices by gradually moving from free-range grazing to indoor stabling. Furthermore, in Sudan, Ahmed and Fawi (2018) showed that 56% of dairy farms produced 2 to 6 t of manure/month and that a large share of that manure was sold.

### Social consequences

Women are found to be excluded from handling milk-related money when this activity becomes economically important in the household (Table 1). Among the Fulani, income from milk goes exclusively to women. However, the situation seems to change once the decision is made to sell the milk to a dairy. This trend is not specific to Burkina Faso and West Africa. In East Africa, Herego (2017) and Umuzigambeho (2017) showed that in milk value chains, women tended to be more focused on home-based production and processing. With the intensification and marketing of dairy products, women’s workloads tend to increase, leading to their being sidelined and to men taking over in the marketing chain. Beyond production, men predominate in the milk value chain as milk dealers, animal healthcare agents, AI service providers and extension staff. Policies introduced in these countries to promote women’s inclusion in value chains have been slow to produce results. Those policies seek, in particular, to strengthen women’s involvement in the management of dairy cooperatives and to improve their access to credit and training.

### Heading for the intensification of milk production

The emergence of market-oriented dairy farmers and agropastoralists suggests that the path towards production intensification followed by those dairy systems, which is based on a stronger crop-livestock combination, improved cattle housing, parsimonious use of local feed concentrates and the introduction of exotic dairy breeds mainly through AI and cross-breeding with local zebus, is broadly consistent with that described by Chagunda et al. (2015) among small-scale dairy farmers in East Africa. We have qualified this path of low-cost intensification driven by market opportunity.

None of the three dairy system classes described in our study fully meets the five criteria for a sustainable livestock system provided by Dumont et al. (2013). According to those criteria, market-oriented farmers’ dairy systems are those showing the most optimised metabolic operation of the livestock system (principle 3), the lowest healthcare expenditure per capita (principle 1) and where domestic feed and fodder production efforts reduce dependency on external inputs (principle 2), but which at the same time make greater use of feeds purchased on the market, tend to specialise production and simplify management methods, which goes against principles 2, 4 and 5 in the grid.

In further work, it would be interesting to look in greater depth at dairy producers’ ecological footprints and agroecological profiles, taking into account, as suggested by Funes-Monzote et al. (2009), indicators that do not refer solely to the production unit (in this case the cow being milked), but also, for example, the yield per asset or per unit area, along with biodiversity indicators and energy balances.

Finally, this study shows that the improvement in dairy systems’ technical and economic performance in Burkina Faso is mainly based on the genetics of the cows making up the operation and on the feed provided to the animals, but also on a better recycling loop for the holding’s agricultural by-products.

The study highlights issues of concern regarding current genetic breeding practices (based on exotic breeds), excessive use of feed concentrates on the more intensively farmed holdings and the sidelining of women from the household milk economy once that activity becomes important.

The low-cost intensification market-oriented trend described in this study must draw the attention of researchers and developers alike to support dairy producers in building sustainable pathways.

## Data Availability

The datasets generated during and/or analysed during the current study are available from the corresponding author on reasonable request.
